# Video-assisted thoracic bronchial sleeve lobectomy with bronchoplasty for treatment of lung cancer confined to a single lung lobe: a case series of Chinese patients

**DOI:** 10.1186/1749-8090-9-67

**Published:** 2014-04-04

**Authors:** Daping Yu, Yi Han, Shijie Zhou, Xiaoyun Song, Yunsong Li, Ning Xiao, Zhidong Liu

**Affiliations:** 1 Department of thoracic surgery, Beijing Chest Hospital, Beijing 101149, China

**Keywords:** Video-assisted, Bronchial sleeve lobectomy, Lung cancer, Squamous carcinoma, Carcinoid, Thoracoscopic lobectomy, VABSL, VATS

## Abstract

**Background:**

The outcomes of video-assisted thoracic bronchial sleeve lobectomy (VABSL), a minimally invasive video-assisted thoracoscopic (VATS) lobectomy, are mostly unknown in Chinese patients.

**Objectives:**

To investigate operative and postoperative outcomes of VABSL in a cases series of Chinese patients with lung cancer.

**Methods:**

Retrospective study of 9 patients (male:female 8:1; mean age 59.4 ± 17.6 years, ranging 21–79 years) diagnosed with lung cancer of a single lobe, treated with VABSL between March 2009 and November 2011, and followed up for at least 2 months (mean follow-up: 14.17 ± 12.91 months). Operative outcomes (tumor size, operation time, estimated blood loss and blood transfusion), postoperative outcomes (intensive care unit [ICU] stay, hospitalization length and pathological tumor stage), death, tumor recurrence and safety were assessed.

**Results:**

Patients were diagnosed with carcinoid cancer (11.1%), squamous carcinoma (66.7%) or small cell carcinoma (22.2%), affecting the right (77.8%) or left (22.2%) lung lobes in the upper (55.6%), middle (11.1%) or lower (33.3%) regions. TNM stages were T2 (88.9%) or T3 (11.1%); N0 (66.7%), N1 (11.1%) or N2 (22.2%); and M0 (100%). No patient required conversion to thoracotomy. Mean tumor size, operation time and blood loss were 2.50 ± 0.75 cm, 203 ± 20 min and 390 ± 206 ml, respectively. Patients were treated in the ICU for 18.7 ± 0.7 hours, and overall hospitalization duration was 20.8 ± 2.0 days. No deaths, recurrences or severe complications were reported.

**Conclusions:**

VABSL surgery is safe and effective for treatment of lung cancer by experienced physicians, warranting wider implementation of VABSL and VATS training in China.

## Background

In modern thoracic surgery, anatomically suitable central lung cancers are commonly treated by sleeve lobectomy, regardless of lung function [1]. However, there has been a wide implementation of video-assisted thoracoscopic (VATS) lobectomy for performing bronchial sleeve lobectomy (VABSL), an approach that is minimally invasive, more technically challenging [2], and with good outcomes [3,4]. In the Unites States and Europe, only 20-30% of all lobectomies are performed using VATS, but this proportion is rapidly rising [2] and new training programs are being implemented [5]. In China, the number of lobectomies performed using VATS remains unknown, as there have been very few published reports in lung cancer patients [6-8].

The use of bronchial anastomosis is the primary feature that distinguishes VABSL from VATS lobectomy [9]. In most cases, bronchial anastomosis is accomplished using an open surgical technique through a mini-thoracotomy with conventional open surgical instruments, although the recent adoption of video-assistance and robotic surgery has improved the outcomes of these procedures [10]. Compared with open lobectomy or thoracotomy, VATS and VABSL have some advantages, including smaller incisions, decreased postoperative pain and shorter hospital stays [9,11]. Thus, VABSL is an effective alternative to thoracotomy for many patients with lung cancer.

The present study retrospectively reviewed nine Chinese lung cancer patients who were treated with VABSL. Our findings provide evidence that VABSL may be effective and safe for Chinese patients with lung cancer by reducing bleeding, hospitalization time and complication rate, compared with conventional open lobectomy and thoracotomy.

## Methods

### Patients

A total of 9 patients, diagnosed with squamous carcinoma, small cell carcinoma, or carcinoid cancer of a single lung lobe, were treated with VABSL between March 2009 and November 2011 at the Beijing Chest Hospital, Beijing, People’s Republic of China (Table 1). No patient had a history of thoracotomy. The study protocol was approved by the Ethics Committee of the Beijing Chest Hospital (Beijing, People’s Republic of China). All patients provided written informed consent.

**Table 1:** Patients included in the study

**No.**	**Age (yr)**	**Sex**	**Diagnosis**	**Site**		**TNM stage**			**Follow-up (mo)**	**Recurrence**
				**Side**	**Position**	**T**	**N**	**M**		
1	56	M	Squamous carcinoma	R	u	2	0	0	22	0
2	70	M	Squamous carcinoma	R	u	2	0	0	3.5	0
3	46	M	Small cell carcinoma	R	l	2	1	0	9	0
4	68	M	Squamous carcinoma	R	u	3	0	0	43	0
5	73	M	Small cell carcinoma	R	u	2	0	0	18	0
6	79	M	Squamous carcinoma	L	l	2	2	0	>3	0
7	56	M	Squamous carcinoma	R	m	2	2	0	11	0
8	66	M	Squamous carcinoma	L	u	2	0	0	>2	0
9	21	F	Carcinoid cancer	R	l	2	0	0	16	0

*Abbreviations*: R, right; L, left; u, upper; m, middle; l, lower.

### Operative procedure

All thoracic surgeons were trained and experienced in VABSL. The decision to perform VABSL was based on surgeons’ preference, intraoperative assessment of adhesions and calcifications adjacent to pulmonary branches, and preoperative computed tomography (CT) [12]. Mediastinoscopy was performed in all patients to exclude a N2 disease.

Patients were placed in prone position. Endoshears/Endokittners (US Surgical, Norwalk, CT, USA) were used for dissection, and thoracic bronchial sleeve lobectomy was performed through four 1-cm thoracic ports. Pulmonary artery and vein branches were divided using an EndoGIA vascular stapler (U.S. Surgical, Norwalk, CT, USA). Bronchus was divided using scissors. Bronchial anastomosis was performed, and lesions were directly palpated through the utility incision without trocar use (except for the camera port). In the early surgeries, discontinuous anastomosis was performed using 3–0 absorbable suture to discontinuously suture the whole layer: the less exposed tracheal wall was sutured and the anterior tracheal wall was anastomosed first, and prepositioned silk was ligated and sutured from the tracheal cartilage. In the late surgeries, continuous anastomosis combined with discontinuous consolidation was performed using 3–0 absorbable suture to first anastomose the less exposed tracheal wall and the anterior tracheal wall. Anastomosis was continuously sutured for about 1/3 of the whole cycle when the two ends of the suture were fixed, followed by continuous suture for 1/3 and 1/3 cycles from the two fixed ends respectively. If an air leak was observed, discontinuous suture and repair were performed using 3–0 absorbable suture.

The entire operation was performed with conventional long instruments, as previously described [9]. Considerations were made for the anatomical position of the lesion for each patient.

Freed lobes were placed in an Endocatch bag (U.S. Surgical, Norwalk, CT, USA). The chest was irrigated, and the anastomosis checked for leakage. Two 28 F chest tubes were placed, and the incisions were closed. Postoperative bronchoscopy was performed to clear blood and secretions from the airways prior to extubation, as well as to observe the healing of the anastomotic stoma and the eventual occurrence of fistula and stenosis [9].

Tumor size, operation time, estimated blood loss and blood transfusion requirement were assessed for each patient.

### Postoperative assessments

Immediately following surgery, each patient was kept in the intensive care unit (ICU) until stable, and then admitted for in-patient recovery. The duration of the ICU stay and the total duration of the hospital stay (as an in-patient) were recorded.

### Follow-up

Patients were followed up for a minimum of 2 months after surgery (mean follow-up: 14.2 ± 12.9 months). All patients underwent at least one clinical examination more than 1 month after surgery. The occurrence of death, tumor recurrence or other adverse events was recorded.

## Results

### Patients’ demographic and clinical characteristics

Patients (N = 9) had a mean age of 59.4 ± 17.6 years, ranging from 21 to 79 years. Most patients (8/9, 88.9%) were male. On final pathological examination, patients were diagnosed with carcinoid cancer (1/9, 11.1%), squamous carcinoma (6/9, 66.7%) or small cell carcinoma (2/9, 22.2%), affecting the right (7/9, 77.8%) or left (2/9, 22.2%) lung lobes. However, the two cases of small cell carcinoma were initially diagnosed as poorly differentiated squamous carcinomas on preoperative biopsy. The lesions were positioned in the upper, middle and lower lobe regions in 5 (55.6%), 1 (11.1%), and 3 (33.3%) patients, respectively. In all patients, preoperative mediastinoscopy indicated an absence of local metastasis. In addition, no patient had a history of neoadjuvant treatments or thoracotomy. Final pathological TNM staging was: T2 (8/9, 88.9%) and T3 (1/9, 11.1%); N0 (6/9, 66.7%), N1 (1/9, 11.1%), and N2 (2/9, 22.2%); and M0 (9/9, 100%) (Tables 1 and 2).

**Table 2:** Baseline demographic and clinical characteristics

**Characteristic**	**Value**
Age (years)*	59.4 ± 17.6 (21–79)
Gender^#^	
Male	8 (88.9%)
Female	1 (11.1%)
Prior thoracotomy^#^	0 (0%)
TNM stage^#^	
T2	8 (88.9%)
T3	1 (11.1%)
N0	6 (66.7%)
N1	1 (11.1%)
N2	2 (22.2%)
M0	9 (100%)
Follow-up time (months)*	14.2 ± 12.9 (2–43)
Diagnosis^#^	
Carcinoid cancer	1 (11.1%)
Squamous carcinoma	6 (66.7%)
Small cell carcinoma	2 (22.2%)
Lung lobe affected^#^	
Right	7 (77.8%)
Left	2 (22.2%)
Upper	5 (55.6%)
Middle	1 (11.1%)
Lower	3 (33.3%)

*Data are presented as the mean ± SD (range).

^#^Data are presented as *n* (%), where *n* is the number of patients (N = 9).

### Operative characteristics

Conversion to open thoracotomy was not required for any patient. Resection of the right upper lung lobe was the most common (4/9, 44.4%), followed by the right lower lobe (2/9, 22.2%). The mean tumor size was 2.50 ± 0.75 cm. The duration of surgery was 203 ± 20 min, with an estimated blood loss of 390 ± 206 ml. Blood transfusions were required in 3 patients (Table 3).

**Table 3:** Operative assessments

**Assessment**	**Value**
Type of resection^#^	
Right upper lobe	4 (44.4%)
Right middle lobe	1 (11.1%)
Right lower lobe	2 (22.2%)
Left upper lobe	1 (11.1%)
Left lower lobe	1 (11.1%)
Tumor size (cm)*	2.50 ± 0.75
Estimated blood loss (ml)*	390 ± 206
Operation time (min)*	203 ± 20
Blood transfusion (ml)*	133.3 ± 200.0

*Data are presented as the mean ± SD.

^#^Data are presented as *n* (%), where *n* is the number of patients (N = 9).

### Histology and final pathologic tumor stage

The most common histological type was squamous carcinoma. Pathological tumor stage was determined to be IB in 6 (66.7%) patients, IIA in 1 (11.1%) patient and IIIA in 2 (22.2%) patients. Stage IB was thus the most common pathological stage (Table 4).

**Table 4:** Pathological and histological tumor stage

**Assessment**	**Value**
Pathological staging	
IA	0 (0%)
IB	6 (66.7%)
IIA	1 (11.1%)
IIB	0 (0%)
IIIA	2 (22.2%)
Histological type	
Carcinoid – typical	1 (11.1%)
Squamous	6 (66.7%)
Small cell carcinoma	2 (22.2%)

Data are presented as *n* (%), where *n* is the number of patients (N = 9).

### Post-operative and follow-up outcomes

Immediately after surgery, patients stayed in the ICU for a mean of 18.7 ± 0.7 hours before being transferred to normal in-patient facilities. The total duration of hospitalization lasted, on average, 20.8 ± 2.0 days. During hospitalization, pain was effectively treated using routine medication, and no complication due to drainage tube placement or removal was observed. No deaths or severe adverse events were reported during hospitalization or the follow-up period. Furthermore, no patient exhibited recurrence or required secondary surgery (Table 5).

**Table 5:** Postoperative and follow-up outcomes

**Assessment**	**Value**
ICU stay (hours)*	18.7 ± 0.7
Hospitalization (days)*	20.8 ± 2.0
Recurrence^#^	0 (0%)
Death^#^	0 (0%)
Adverse events^#^	0 (0%)

*Data are presented as the mean ± SD.

^#^Data are presented as *n* (%), where *n* is the number of patients (N = 9).

## Discussion

VABSL was successfully used to treat lung cancer without short-term occurrence of metastasis, cancer recurrence, or death in nine patients. Furthermore, all complications and adverse events (such as pain) were mild, and were treated using routine medications. In addition, bleeding and hospitalization duration were minimal, with no cases of excessive bleeding or drainage. Thus, VABSL may be a valuable, minimally invasive method for the treatment of many different forms of lung cancer in Chinese patients, although adequate thoracic surgeon training and experience may be required to achieve optimal results.

There are controversies about the complication rates associated with the use of VABSL, and how these rates compare with those of open surgery. Indeed, various studies worldwide have reported highly variant hospitalization requirements and costs. In a study conducted in the United States, Mahtabifard et al. [9] reported that a large proportion of patients undergoing VABSL experienced severe complications (15.4%), including edema, stricture of the anastomosis site requiring airway stenting, and anastomotic dehiscence. Similarly, Belgers et al. [13] reported that 10% of 172 patients treated with VATS in the Netherlands experienced complications. A study in Spain also reported a similar complication rate (12.8%), mostly due to air leakage [14]. Conversely, a study of 362 lung resection cases in Japan using VABSL reported a median postoperative hospital stay of only seven days, without major postoperative complications [15]. A single-center Israeli study conducted by Arad et al. [16] suggested that the complication rate decreased after the first 6 months of VATS implementation, indicating that surgeon’s experience was involved in obtaining good outcomes.

In the present study, no severe complications were observed and the mean hospitalization duration was 20.8 ± 2.0 days. These discrepancies may be due to the selection of the operative field [15] or experience with the technique [16]. Furthermore, video-assisted surgery for lung cancer has been shown to produce dramatically shorter hospitalization duration than conventional open surgery [14]. These findings were confirmed on a much larger scale by the American College of Surgeons Oncology Group Z0030 trial that included 964 participants, and demonstrated that the majority of patients treated with VATS exhibited shorter hospital stays and fewer complications than patients undergoing open surgery [17]. Although VATS and VABSL have been less studied in China, Li and Wang have published two reports describing the successful use of VABSL with bronchoplasty in Chinese patients, with only one patient in their cohort of 10 [7] and 15 [8] experiencing minor complications. A recent review by Zhang et al. [18] suggested that video-assisted thoracic lobectomy may reduce cancer recurrence and result in better overall survival compared with conventional open thoracotomy for non-small-cell lung cancer patients. Thus, VABSL is clearly superior to open surgery for eligible patients, and is increasingly being implemented worldwide as a treatment for lung cancer. However, better standardization will be required as VATS and VABSL become increasingly implemented as routine lung cancer strategies, in order to achieve better consistency of treatment outcomes.

One of the central benefits of VATS surgery over open lobectomy is that bleeding is minimized, thereby reducing fluid drainage and subsequent complications involving edema or bleeding in the pleural cavity [19]. In fact, VATS has been suggested as a tool for alleviation of bleeding in other conditions, such as spontaneous hemopneumothorax, due to its effectiveness at not only reducing, but also eliminating bleeding in the pleural cavity [20]. In the study by Belgers et al. [13], which reported a relatively high complication rate for VATS, the mean blood loss was 444 ml and the mean operating time was 179 min. It is important to note that the lower level of bleeding in the present study (390 ± 206 ml) may have contributed to the overall good outcomes and low complication rate. These findings may also be related to the comparatively short mean operating time of 203 ± 20 min, which may be the result of more experienced or better trained surgeons. However, when accounting for the time lost for adjusting surgical focus, stabilization and modulation of the monitors, our patients were in the operating room for 292 ± 46 min. Although bleeding may vary between VATS methods, a recent meta-analysis reported that bleeding was not significantly different between open surgery and VATS. However, in each case, mortality, morbidity, air leakage, pneumonia, sepsis and atrial arrhythmias were reduced in patients receiving VATS compared with those who underwent open surgery. Although the amount of bleeding caused by VABSL is highly variable, the overall outcomes are consistently better than those observed with open surgery.

Although discontinuous anastomosis is reliable, the suture was often ligated in the tracheal lumen, which caused postoperative sputum stagnation and irritable cough. In addition, since the handle hole under the thoracoscope is limited, the operation was more difficult with longer surgical time. In contrast, continuous anastomosis combined with discontinuous consolidation avoided the presence of absorbable suture in the tracheal lumen, therefore decreasing postoperative sputum stagnation and irritable cough; it also decreased the anastomosis time.

In general, lower mortality has been observed in patients undergoing modern VABSL procedures compared with those undergoing conventional open surgery. Loscertales et al. [14], using 17 recent studies, concluded that patients undergoing VATS lobectomy experienced less postoperative pain, reduced chest tube time, and overall better outcomes, including reduced mortality, than open surgery patients. Mahtabifard et al. [9] also reported lower mortality in patients undergoing VABSL. Furthermore, a meta-analysis of 21 studies (2 randomized and 19 non-randomized) indicated that VATS surgery resulted in lower 5-year all-cause mortality, as well as lower recurrence rates [21], consistent with the low mortality and recurrence observed in the present study. Cumulatively, the present findings and previous research indicate that VABSL provides superior long-term outcomes and limits lung cancer recurrence, perhaps even more effectively than conventional open surgery. Thus, we recommend the wider implementation of VABSL in China in patients with lung cancer who are deemed suitable for lobectomy with bronchoplasty.

The evidence-based clinical practice guidelines of the American College of Chest Physicians recently suggested that “In patients with stage I NSCLC who are considered appropriate candidates for thoracoscopic anatomic lung resection (lobectomy or segmentectomy), the use of VATS by surgeons experienced in these techniques is an acceptable alternative to open thoracotomy” [9], consistent with our current recommendations. While operator experience is a key factor to surgical success of VATS and VABSL, guidelines for junior surgeons (<100 surgeries performed) have been recently proposed in Korea and indicate that the VATS lobectomy technique can be dramatically improved over the course of only 6 months. Thereafter, junior surgeons reported similar conversions to open surgery and outcomes as experienced surgeons [22]. Similarly, European VATS training programs have been designed, and showed that prolonged air leak, chest tube duration, operation time and length of stay were reduced by training, most likely due to superior patient selection by trained operators [23,24]. As VATS and VABSL are technically challenging procedures that have been demonstrated to be more successful when performed by specially-trained thoracic surgeons, it has been suggested that training for these procedures should be coordinated at a national level as they become more widely implemented [24]. It may be beneficial to consider implementing similar national training and patient eligibility determination in China, though further research will be required to determine the optimal parameters in Chinese clinical practice settings.

It is important to consider that the present results were obtained from a relatively small and non-diverse patient population of only 9 predominantly male cases. Thus, these findings may not be typical of the general population of Chinese lung cancer patients. Similarly, the surgeons in the present study were both trained and experienced, and less qualified surgeons may experience poorer results and a greater need for intraoperative conversion to open surgery, further highlighting the need for additional standardization and regulation of training in VATS techniques in China. Additionally, VABSL may be less effective for more advanced cancer stages where lymph nodes infiltration has occurred. Further research should be conducted to validate these findings in larger clinical populations with more diverse presentations of lung cancer.

## Conclusions

The present results provide preliminary evidence that VABSL is both safe and effective for the treatment of lung cancer in Chinese patients, consistent with previous reports worldwide. Furthermore, VABSL may minimize complication rates when used by trained and experienced thoracic surgeons, resulting in less bleeding, lower recurrence rates, and better overall outcomes than conventional open lobectomy surgery. Thus, we recommend that VATS and VABSL should be more widely implemented in China, along with appropriate, nationally-regulated training programs.

## Abbreviations

VABSL: Video-assisted thoracic bronchial sleeve lobectomy; VATS: Video-assisted thoracoscopic; ICU: Intensive care unit; CT: Computed tomography.

## Competing interest

The authors declare no competing of interest.

## Author contributions

All authors contributed to preparation of the manuscript, and read and approved the final version.

## References

[B1] Wright CD Sleeve lobectomy in lung cancer Seminars in Thoracic and Cardiovascular Surgery 2006 Amsterdam: Elsevier 92 95 10.1053/j.semtcvs.2006.05.003 17157226

[B2] Boffa DJ Allen MS Grab JD Gaissert HA Harpole DH Wright CD Data from The Society of Thoracic Surgeons General Thoracic Surgery database: the surgical management of primary lung tumors J Thorac Cardiovasc Surg 2008 9 247 254 10.1016/j.jtcvs.2007.07.060 18242243

[B3] McKenna RJ Jr Houck W Fuller CB Video-assisted thoracic surgery lobectomy: experience with 1,100 cases Ann Thorac Surg 2006 9 421 425 discussion 425–426 10.1016/j.athoracsur.2005.07.078 16427825

[B4] Seong YW Yoo BS Kim JT Park IK Kang CH Kim YT Video-assisted thoracoscopic lobectomy in children: safety and efficacy compared with the conventional thoracotomy approach Innovations 2012 9 394 398 23422800 10.1177/155698451200700604

[B5] Bille A Okiror L Karenovics W Choudhuri D Routledge T Thoracoscopic lobectomy: is a training program feasible with low postoperative morbidity? Gen Thorac Cardiovasc Surg 2013 9 409 413 10.1007/s11748-013-0225-5 23456987

[B6] Jiao W Zhao Y Huang T Shen Y Two-port approach for fully thoracoscopic right upper lobe sleeve lobectomy J Cardiothorac Surg 2013 9 99 10.1186/1749-8090-8-99 23594432 PMC3639094

[B7] Li Y Wang J Video-assisted thoracoscopic surgery sleeve lobectomy with bronchoplasty World J Surg 2013 9 1661 1665 10.1007/s00268-013-2032-7 23571864

[B8] Li Y Wang J Video-assisted thoracoscopic surgery sleeve lobectomy with bronchoplasty: an improved operative technique Eur J Cardiothorac Surg 2013 9 1108 1112 10.1093/ejcts/ezt199 23557917

[B9] Mahtabifard A Fuller CB McKenna RJ Jr Video-assisted thoracic surgery sleeve lobectomy: a case series Ann Thorac Surg 2008 9 S729 S732 10.1016/j.athoracsur.2007.12.001 18222205

[B10] Schmid T Augustin F Kainz G Pratschke J Bodner J Hybrid video-assisted thoracic surgery-robotic minimally invasive right upper lobe sleeve lobectomy Ann Thorac Surg 2011 9 1961 1965 10.1016/j.athoracsur.2010.08.079 21619991

[B11] Hoksch B Ablassmaier B Walter M Muller JM Complication rate after thoracoscopic and conventional lobectomy Zentralblatt fur Chirurgie 2003 9 106 110 10.1055/s-2003-37763 12632277

[B12] Luketich JD Meehan MA Landreneau RJ Christie NA Close JM Ferson PF Keenan RJ Belani CP Total videothoracoscopic lobectomy versus open thoracotomy for early-stage non small-cell lung cancer Clin Lung Cancer 2000 9 56 60 discussion 61 10.3816/CLC.2000.n.018 14731340

[B13] Belgers EH Siebenga J Bosch AM van Haren EH Bollen EC Complete video-assisted thoracoscopic surgery lobectomy and its learning curve. A single center study introducing the technique in The Netherlands Interact Cardiovasc Thorac Surg 2010 9 176 180 10.1510/icvts.2009.212878 19850598

[B14] Loscertales J Quero Valen Zuela F Congregado M Jimenez Merchan R Gallardo Varela G Trivino Ramirez A BMM S Cozar Bernal F Video-assisted thoracic surgery lobectomy: results in lung cancer J Thorac Dis 2010 9 29 35 22263014 PMC3256439

[B15] Kamiyoshihara M Nagashima T Igai H Atsumi J Ibe T Kakegawa S Shimizu K Video-assisted thoracic lobectomy with bronchoplasty for lung cancer, with special reference to methodology Interact Cardiovasc Thorac Surg 2011 9 534 538 10.1510/icvts.2010.258228 21266490

[B16] Arad T Levi-Faber D Nir RR Kremer R The learning curve of video-assisted thoracoscopic surgery (VATS) for lung lobectomy--a single Israeli center experience Harefuah 2012 9 261 265 320 22844727

[B17] Scott WJ Allen MS Darling G Meyers B Decker PA Putnam JB McKenna RW Landrenau RJ Jones DR Inculet RI Malthaner RA Video-assisted thoracic surgery versus open lobectomy for lung cancer: a secondary analysis of data from the American College of Surgeons Oncology Group Z0030 randomized clinical trial J Thorac Cardiovasc Surg 2010 9 976 981 discussion 981–973 10.1016/j.jtcvs.2009.11.059 20172539

[B18] Zhang Z Zhang Y Feng H Yao Z Teng J Wei D Liu D Is video-assisted thoracic surgery lobectomy better than thoracotomy for early-stage non-small-cell lung cancer? A systematic review and meta-analysis Eur J Cardiothorac Surg 2013 9 407 414 10.1093/ejcts/ezt015 23371973

[B19] Dylewdki MR Lazzaro RS Robotics - The answer to the Achilles' heel of VATS pulmonary resection Chin J Cancer Res 2012 9 259 260 10.1007/s11670-012-0261-1 23359547 PMC3551333

[B20] Luh SP Tsao TC Video-assisted thoracic surgery for spontaneous haemopneumothorax Respirology 2007 9 443 447 10.1111/j.1440-1843.2006.01008.x 17539853

[B21] Yan TD Black D Bannon PG McCaughan BC Systematic review and meta-analysis of randomized and nonrandomized trials on safety and efficacy of video-assisted thoracic surgery lobectomy for early-stage non-small-cell lung cancer J Clin Oncol 2009 9 2553 2562 10.1200/JCO.2008.18.2733 19289625

[B22] Ra YJ Ahn HY Kim MS Learning Curve of a Young Surgeon’s Video-assisted Thoracic Surgery Lobectomy during His First Year Experience in Newly Established Institution Korean J Thorac Cardiovasc Surg 2012 9 166 170 10.5090/kjtcs.2012.45.3.166 22708084 PMC3373972

[B23] Petersen RH Hansen HJ Learning thoracoscopic lobectomy Eur J Cardiothorac Surg 2010 9 516 520 10.1016/j.ejcts.2009.09.012 19818641

[B24] Ferguson J Walker W Developing a VATS lobectomy programme–can VATS lobectomy be taught? Eur J Cardiothorac Surg 2006 9 806 809 10.1016/j.ejcts.2006.02.012 16581257

